# Negative Correlation Learning for Customer Churn Prediction: A Comparison Study

**DOI:** 10.1155/2015/473283

**Published:** 2015-03-23

**Authors:** Ali Rodan, Ayham Fayyoumi, Hossam Faris, Jamal Alsakran, Omar Al-Kadi

**Affiliations:** ^1^King Abdulla II School for Information Technology, The University of Jordan, Amman 11942, Jordan; ^2^College of Computer and Information Sciences, Al Imam Mohammad Ibn Saud Islamic University, Riyadh 11432, Saudi Arabia

## Abstract

Recently, telecommunication companies have been paying more attention toward the problem of identification of customer churn behavior. In business, it is well known for service providers that attracting new customers is much more expensive than retaining existing ones. Therefore, adopting accurate models that are able to predict customer churn can effectively help in customer retention campaigns and maximizing the profit. In this paper we will utilize an ensemble of Multilayer perceptrons (MLP) whose training is obtained using negative correlation learning (NCL) for predicting customer churn in a telecommunication company. Experiments results confirm that NCL based MLP ensemble can achieve better generalization performance (high churn rate) compared with ensemble of MLP without NCL (flat ensemble) and other common data mining techniques used for churn analysis.

## 1. Introduction

Technological improvements have enabled data driven industries to analyze data and extract knowledge. Data mining techniques facilitate the prediction of certain future behavior of customers [1]. One of the most important issues that reduces profit of a company is customer churn, which is also known as customer attrition or customer turnover [2]. Customer churn can also be defined as the business intelligence process of finding customers that are about to switch from a business to its competitor [3].

In todays industries, abundance of choices helps customers get advantage of a highly competitive market. One can choose a service provider that offers better service than others. Therefore, the profitmaking organizations which compete in saturated markets such as banks, telecommunication and internet service companies, and insurance firms strongly focused more on keeping current customers than acquiring new customers [4]. Moreover, maintaining current customers is proven to be much less expensive than acquiring new customers [5].

In order to keep their customers, companies need to have a deep understanding of why churn happens. There are several reasons to be addressed, such as dissatisfaction from the company, competitive prices of other companies, relocation of the customers, and customers' need for a better service which can lead customers to leave their current service provider and switch to another one [6].

Among the previous studies for churn analysis, one of the most frequently used method is artificial neural networks (ANNs). In order to fine-tune the models developed, several topologies and techniques were investigated with ANNs, such as building medium-sized ANN models which were observed to perform the best and making experiments in many domains such as pay-TV, retail, banking, and finance [7]. These studies indicate that a variety of ANN approaches can be applied to increase prediction accuracy of customer churn. In fact, the use of neural networks in churn prediction has a big asset in respect of other methods used, because the likelihood of each classification made can also be determined. In neural networks each attribute is associated with a weight and combinations of weighted attributes participate in the prediction task. The weights are constantly updated during the learning process. Given a customer dataset and a set of predictor variables the neural network tries to calculate a combination of the inputs and to output the probability that the customer is a churner.

On the other hand, data collected for churn prediction is usually imbalanced, where the instances in the nonchurner customer outnumber the instances in the churner class. This is considered as one of the most challenging and important problems since common classification approaches tend to get good accuracy results for the large classes and ignore the small ones [8]. In [9], the authors discussed different approaches for handling the problem of imbalanced data for churn prediction. These approaches include using more appropriate evaluation metrics, using cost-sensitive learning, modifying the distribution of training examples by sampling methods, and using Boosting techniques. In [8], authors added ensemble classifiers as a fourth category of approaches for handling class imbalance. It has been shown that ensemble learning can offer a number of advantages over a single learning machine (e.g., neural network) training. Ensemble learning has a potential to improve generalization and decrease the dependency on training data [10].

One of the key elements for building ensemble models is the “diversity” among individual ensemble members. Negative correlation learning (NCL) [11] is an ensemble learning technique that encourages diversity explicitly among ensemble members through their negative correlation. However, few studies addressed the impact of diversity on imbalanced datasets. In [12], authors indicated that NCL brings diversity into ensemble and achieve higher recall values for minority class comparing NCL to independent ANNs.

Motivated by these possible advantages of NCL for class imbalance problems, in this paper, we apply the idea of NCL to an ensemble of multilayer perceptron (MLP) and investigate its application for customer churn prediction in the telecommunication market. Each MLP in the ensemble operates with a different network structure, possibly capturing different features of the input stream. In general, the individual outputs for each MLP of the ensemble are coupled together by a diversity-enforcing term of the NCL training, which stabilizes the overall collective ensemble output.

Moreover, the proposed ensemble NCL approach will be assessed using different evaluation criteria and compared to conventional prediction techniques and other special techniques proposed in the literature for handling class imbalance cases.

The paper has the following organization.  Section 2 gives a background on data mining techniques used in the literature for churn analysis. In Section 3 we introduce negative correlation learning and how to use it to generate an ensemble of MLP with “diverse” members. Churn dataset description is given in Section 4. Experiments and results are presented in Section 5. Finally, our work is concluded in Section 6.

## 2. Related Work

Data mining techniques that are used in both researches and real-world applications generally treat churn prediction as a classification problem. Therefore, the aim is to use past customer data and classify current customers into two classes, namely, prediction churn and prediction nonchurn [7]. There have also been made a few academic studies on clustering and association rule mining techniques.

After having done the necessary data collection and data preprocessing tasks and labeling the past data, features that are relevant to churn need to be defined and extracted. Feature selection, also known as dimension reduction, is one of the key processes in data mining and can alter the quality of prediction dramatically. Preprocessing and feature selection are common tasks that are applied before almost every data mining technique. There are different widely used algorithms for churn analysis, for instance, decision trees; by its algorithm nature, the obtained results can easily be interpreted and therefore give the researcher a better understanding of what features of customer are related to churn decision. This advantage has made decision trees one of the most used methods in this field. Some applications of decision trees in churn prediction include building decision trees for all customers and building a model for each of the customer segments [13]. Another relatively new classification algorithm is support vector machine (SVM). It is widely used in data mining applications, particularly in complex situations. SVM algorithm is proven to outperform several other algorithms by increasing the accuracy of classification and prediction of customers who are about to churn [14, 15].

Some other algorithms might be appropriate for customer churn prediction, as the artificial neural networks (ANN), which is another supervised classification algorithm that is used in predicting customer turnover. However, this algorithm is expected to give more accurate results when used in a hybrid model together with other algorithms or with another ANN classifier [16]. Another example could be genetic programming (GP). Genetic programming is an evolutionary method for automatically constructing computer program. These computer programs could be classifiers or regressors represented as trees. The authors in [17] used a GP based approach for modeling a churn prediction problem in telecommunication industry.

Moreover, Bayesian classification is one of the techniques which was also mentioned to be used in churn prediction. This method depends on calculating probabilities for each feature and its effect on determining the class value, which is the customer being a churner or not. However, Bayesian classification may not give satisfactory results when the data is highly dimensional [18].

Lastly, it is worth to mention *k*-nearest neighbor (*k*-NN) and random forests as another two classification methods applied in literature for churn prediction. *k*-NN is a classification method where an instance is classified by a majority vote of its neighbors, where, on the other hand, Random forests are an ensemble of decision trees which are generated from the bootstrap samples. The authors in [19] applied both *k*-NN and random forests to evaluate the performance on sampled and reduced features churn dataset. For some of related work for churn prediction methods, see Table 1.

## 3. Negative Correlation Learning (NCL)

NCL has been successfully applied to training multilayer perceptron (MLP) ensembles in a number of applications, including regression problems [20], classification problems [21], or time series prediction using simple autoregressive models [11].

**Figure 1:**
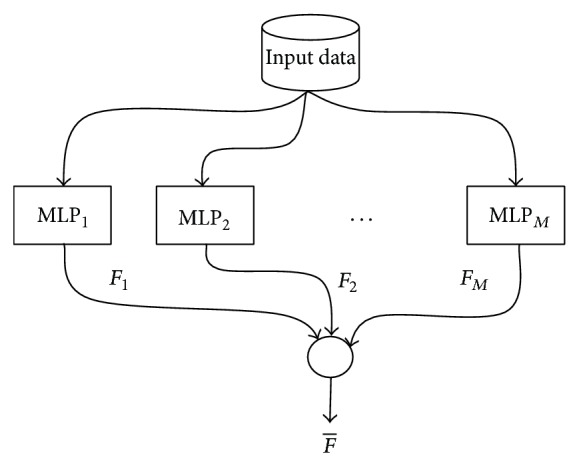
Ensemble of MLP networks.

In NCL, all the individual networks are trained simultaneously and interactively through the correlation penalty terms in their error functions. The procedure has the following form. Given a set of *M* networks and a training input set *s*, the ensemble output *F*(*t*) is calculated as a flat average over all ensemble members (see Figure 1) *F*_*i*_(*t*):

(1) $$\begin{array}{c} F\left(t\right)=\frac{1}{M}\overset{M}{\underset{i=1}{\sum }}‍\left(F_{i}\left(t\right)\right). \end{array}$$

In NCL the penalised error function to be minimised reads as follows:

(2) $$\begin{array}{c} E_{i}=\frac{1}{2}{\left(F_{i}\left(t\right)-y\left(t\right)\right)}^{2}+\lambda p_{i}\left(t\right), \end{array}$$

where

(3) $$\begin{array}{c} p_{i}\left(t\right)=\left(F_{i}\left(t\right)-F\left(t\right)\right)\underset{i\neq j}{\sum }‍\left(F_{j}\left(t\right)-F\left(t\right)\right), \end{array}$$

and *λ* > 0 is an adjustable strength parameter for the negative correlation enforcing penalty term *p*_*i*_. It can be shown that

(4) $$\begin{array}{c} E_{i}=\frac{1}{2}{\left(F_{i}\left(t\right)-y\left(t\right)\right)}^{2}-\lambda {\left(F_{i}\left(t\right)-F\left(t\right)\right)}^{2}. \end{array}$$

Note that when *λ* = 0, we obtain a standard decoupled training of individual ensemble members. Standard gradient-based approaches can be used to minimise *E* by updating the parameters of each individual ensemble member.

### 3.1. Ensembles of MLPs Using NCL

Negative correlation learning (NCL) has been successfully applied to training MLP ensembles [10, 11, 20, 21]. We apply the idea of NCL to the ensemble of multilayer perceptron (MLPs) for predicting customer churn in a telecommunication company. Each MLP neural network operates with a different hidden layer, possibly capturing different features of the customer churn data. Crucially, the individual trained weights of the ensemble are coupled together by a diversity-enforcing term of the NCL training, which stabilises the overall collective ensemble output.

## 4. Dataset Description

The data used in this work is provided by a major Jordanian cellular telecommunication company. The data set contains 11 attributes of randomly selected 5000 customers for a time interval of three months. The last attribute indicates whether the customer churned (left the company) or not. The total number of churners is 381 (0.076 of total customers). The attributes along with their description are listed in Table 2.

The data is normalized by dividing each variable by its standard deviation. Normalization is recommended when data variables follow different dynamic ranges. Therefore, to eliminate the influence of larger values, normalization is applied to make all variables lie in the same scale.

### 4.1. Evaluation Criteria

In order to assess the developed model and compare it with different data mining techniques used for churn analysis, we use the confusion matrix shown in Table 3 which is the primary source for evaluating classification models. Based on this confusion matrix, the following three different criteria are used for the evaluation:

(1) accuracy: measuring the rate of the correctly classified instances of both classes,
  (5) $$\begin{array}{c} \text{Accuracy}=\frac{\left(tp+tn\right)}{\left(tp+fn+fp+tn\right)}, \end{array}$$
(2) hit rate: measuring the rate of predicted churn in actual churn and actual nonchurn,
  (6) $$\begin{array}{c} \text{Hit}\;\text{rate}=\frac{tn}{\left(fn+tn\right)}, \end{array}$$
(3) actual churn rate: measuring the rate of predicted churn in actual churn,
  (7) $$\begin{array}{c} \text{Churn}\;\text{rate}=\frac{tn}{\left(fp+tn\right)}. \end{array}$$

## 5. Experiments and Results

### 5.1. Experimental Setup

In literature, some authors studied the effect of ensemble size on the performance. For example Hansen and Salamon in [22] suggested that ensembles with a small size as ten members were adequate to sufficiently reduce test-set error [23]. In our current paper we used empirical approach to investigate the appropriate size of the ensemble. For ensemble of networks, we tried (4,6, 8,10,12,…, 20) networks in the hidden layer and then checked the performance each time. The best performance reached without overfitting was for an ensemble of size of 10 networks. Therefore, the ensemble used in all our experiments consists of *M* = 10 MLP networks. In all experiments we use MLPs with hidden layer of *N* = 10 units. We used NCL training via backpropagation learning algorithm (BP) on *E* with learning rate *η* = 0.3. The output activation function of the MLP is sigmoid logistic.

We optimize the penalty factor *λ* and the number of hidden nodes using 5-fold cross validation, and *λ* is varied in the range [0,1] (step size 0.1) [10]. The number of hidden nodes is varied from 1 to 20 (step 1). Based on 5-fold cross validation, the details of the selected ensembles of MLPs using NCL parameters are presented in Table 4. Note that the selected parameters for the flat ensembles of MLPs (without NCL) are the same as with NCL except that there are no NCL *λ* parameters. The ensembles used in our experiments are also compared with the following common data mining techniques used in the literature:

(i) *k*-nearest neighbour (IBK),
(ii) Naive Bayes (NB),
(iii) random forest (RF),
(iv) genetic programming,
 (v) single MLP neural network trained with BP,
(vi) C4.5 decision trees algorithm,
(vii) support vector machine (SVM).

As churn data is imbalanced, NCL is also compared with other special techniques proposed in the literature for handling class imbalance cases. These techniques include:

- AdaBoost algorithm with C4.5 Decision Tree as base classifier (AdaBoost), [24].
- Bagging algorithm with C4.5 Decision Tree as base classifier (Bagging) [25],
- MLP for cost-sensitive classification (NNCS) [26],
- synthetic minority oversampling technique with MLP as base classifier (SMOTE + MLP) [27],
- neighborhood cleaning rules with constricted particle swarm optimization as base classifier (NCR + CPSO) [28–30],
- neighborhood cleaning rules with MLP as base classifier (NCR + MLP) [28].

Table 5 presents the empirical settings of the selected models parameters of these common data mining techniques based on 5-fold cross validation. For SVM, cost and gamma parameters are tuned using simple grid search. In C4.5 algorithm, the attributes are computed using the discriminant *P*(*ω*_0_∣*A* = 0) where *P*(·) is the conditional probability and *ω*_*i*_ is the classification type. For naive Bayesian, the transcendent is *p*(*x*∣*w*_*i*_)*P*(*w*_*i*_) − *p*(*x*∣*w*_*j*_)*P*(*w*_*j*_) = 0 where *w*_*i*_ and *w*_*j*_ are the classification types, *p*(*x*∣*w*) is the condition probability density, and *P*(*w*) is the transcendent probability.

### 5.2. Results and Comparison

Table 6 summarizes the results of negatively correlated ensemble of MLPs, independent ensemble of MLPs (*λ* = 0), and other data mining models used in the literature for customer churn prediction. In order to assess the improvement achieved by using a genuine NCL training against independent training of ensemble members (*λ* = 0), the MLP networks are initialized with the same weight values in both cases (see Table 4). The MLP ensemble trained via NCL outperformed independent ensemble of MLPs model and all the other models used in this study.

Note that the two MLP ensemble versions we study share the same number of free parameters, with the sole exception of the single diversity-imposing parameter *λ* in NCL based learning.

According to Table 6, it can be noticed that NCL achieved high accuracy rate (97.1%) and ranked among the best five techniques (results are shown in bold font) with a slight difference (i.e, 0.6%) behind the best which is SVM. The flat ensemble ANN comes after with 95.8% accuracy. As mentioned earlier, accuracy is not the best evaluation metric when the examined problem is highly imbalanced. Therefore, we need to check other criteria; in our case, they are the churn rate and the hit rate. Looking at the obtained churn rates, NCL comes second with 80.3% churn rate and significant increase of 10% over SVM. It is important to indicate here that although NB is the best in churn rate with 90.1%, it is the 2nd worst in terms of accuracy rate which is 59.7%. This gives a great advantage of NCL over NB. Finally, the hit rates of IBK, NB, RF, GP, NNCS, SMOTE + MLP, and NCR + MLP show very poor results so they can be knocked off the race. On the other hand, NCL and ensemble ANN show acceptable results for hit rate of 81.4% and 72.5%, respectively.

## 6. Conclusion

Customer churn prediction problem is important and challenging at the same time. Telecommunication companies are investing more in building accurate churn prediction model in order to help them in designing effective customer retention strategies. In this paper we investigate the application of an ensemble of multilayer perceptron (MLPs) trained through negative correlation learning (NCL) for predicting customer churn in a telecommunication company. Experimental results confirm that NCL ensembles achieve better generalization performance in terms of churn rate prediction with highly acceptable accuracy error rate compared with flat ensembles of MLPs and other common machine learning models from the literature.

## Figures and Tables

**Table 1:** Related work for churn prediction methods.

Author	Method	Description
Idris et al. [17]	GP	GP is applied with AdaBoost for churn prediction

Tsai and Lu [16]	ANN with BP	Applied as a hybrid approach in two stages (i.e., reduction and prediction)

Wang and Niu [14]	SVM	Least squares support vector machine (LS-SVM) is applied to establish a prediction model of credit card customer churn

Eastwood and Gabrys [31]	IBK	Authors apply simple *k*-nearest neighbor algorithm on a nonsequential representation of sequential data for churn prediction

Kraljevíc and Gotovac [32]	Decision trees	Decision trees (DT) were applied and compared with ANN and logistic regression. DT results outperform other models

Verbraken et al. [33]	Naive Bayes	Number of Bayesian Network algorithms, ranging from the Naive Bayes classifier to General Bayesian Network classifiers are applied for churn prediction

**Table 2:** List of attributes.

Attribute name	Description
3G	Subscriber is provided with 3G service (yes, no)
Total consumption	Total monthly fees (calling + sms) (JD)
Calling fees	Total monthly calling fees (JD)
Local sms fees	Monthly local sms fees (JD)
International sms fees	Monthly fees for international sms (JD)
International calling fees	Monthly fees for international calling (JD)
Local sms count	Number of monthly local sms
International sms count	Number of monthly international sms
International MOU	Total of international outgoing calls in minutes
Total MOU	Total minutes of use for all outgoing calls
On net MOU	Minutes of use for on-net-outgoing calls
Churn	Churning customer status (yes, no)

**Table 3:** Confusion matrix.

	Predicted	
	Nonchurn	Churn
Actual nonchurn	*tp*	*fn*
Actual churn	*fp*	*tn*

**Table 4:** Selected ensemble of MLP using NCL parameters based on 5-fold cross validation.

Parameter	Value
Hidden layers	1
Ensemble size (*M*)	10
Decay	0.001
hidden Layer nodes (*N*)	10
Activation function	1/(1 + *e*(−*x*))
Learning rate (*η*)	0.3
Momentum	0.2
*λ*	0.5

**Table 5:** Tuning parameters for data mining techniques used in the comparison study.

Method	Parameters
GP	Population size = 1000, Maximum number of generations = 100, functions = {∗, /, −, +, IF, <, >}, tree max depth = 10, tree max length = 30 elites = 1, selection mechanism = tournament selection crossover point probability = 90%, mutation = probability 15%

ANN with BP	Activation function = Sigmoid, Epoches = 5000, Learning Rate = 0.3, Momentum = 0.2

SVM	Cost = 1, Gamma = 10000

IBK	Number of neighbours = 1, nearest neighbor search algorithm = linear search (brute force search)

AdaBoost	Number of classifiers = 10

Bagging	Number of classifiers = 10

NNCS	Hidden layers = 2, hidden nodes = 15

SMOTE	Number of neighbors = 5

NCR + CPSO	Number of neighbors = 5 for SMOTE, number of particles = 75 for CPSO

**Table 6:** Evaluation results (results of best five models are shown in bold).

Model	Accuracy	Actual churn rate	Hit rate
*k*-Nearest neighbour (IBK)	0.927	0.022	0.067
Naive Bayes (NB)	0.597	0.901	0.115
Random Forest (RF)	0.940	0.006	0.109
Genetic programing (GP)	0.759	0.638	0.142
Single ANN with BP	0.941	0.625	0.607
Decision tress (C4.5)	**0.975**	0.703	**0.964 **
Support vector machine (SVM)	**0.977**	0.703	**0.992 **
AdaBoosting	**0.972**	0.719	**0.898**
Bagging	**0.975**	0.703	**0.954**
MLP for cost-sensitive classification (NNCS)	0.496	0.819	0.113
SMOTE + MLP	0.722	0.724	0.177
NCR + CPSO	0.894	0.827	0.694
NCR + MLP	0.642	0.751	0.144
Flat ensemble of ANN	0.958	0.732	0.725
Ensemble of ANN using (NCL)	**0.971**	**0.803 **	**0.814**
